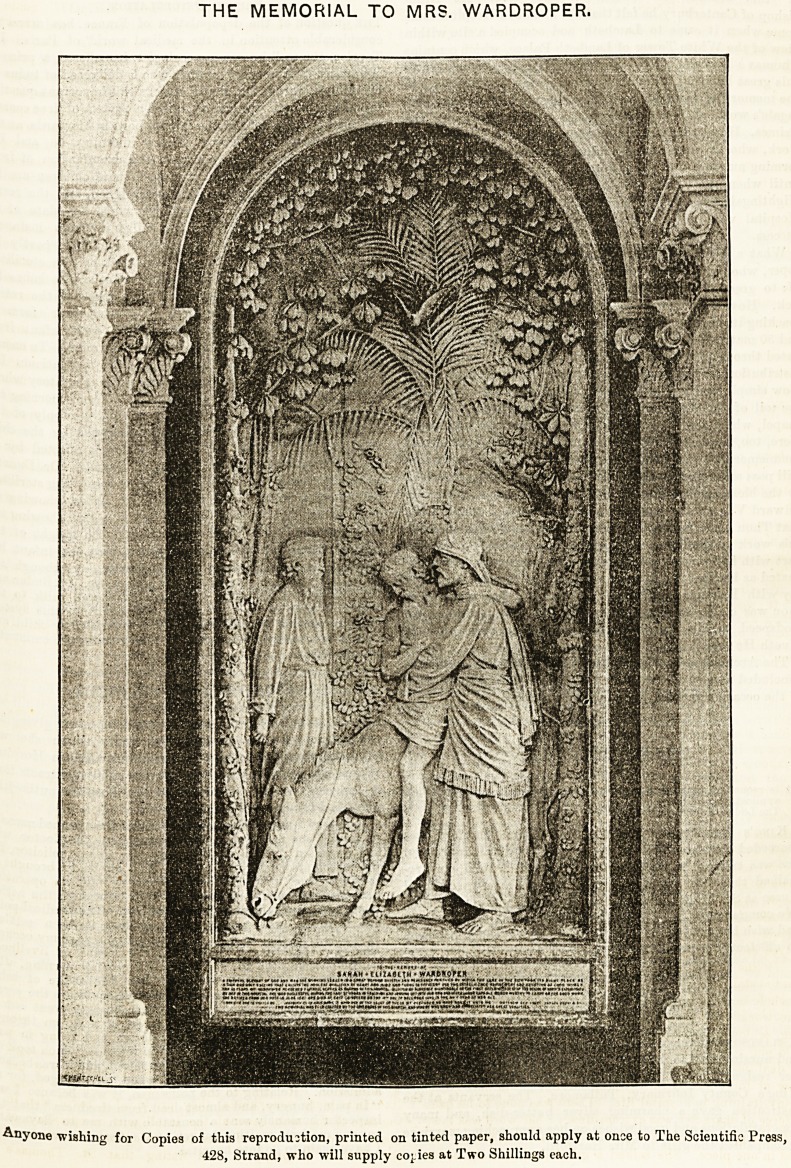# The Hospital Nursing Supplement

**Published:** 1894-05-05

**Authors:** 


"The Hospital" Nursing Mirror.

Being the Extra Nursing Supplement of "The Hospital" Newspaper.

[Contributions for this Supplement should be addressed to the Editor, The Hospital, 428, Strand, London, W.C., and should have the word "Nursing" plainly written in left-hand top corner of the envelope.]

### News from the Nursing World.

### "THE HOSPITAL" CONVALESCENT FUND.

Since the day when the idea of maintaining a free bed for nurses was first laid before our readers The Hospital has grown in size and in importance annually. Therefore we have good reason for hoping that the Fund will also grow in due proportion and a larger number of nurses will each year be enabled to secure the rest they need. In accordance with suggestions made to us we shall no longer have the nurses' free bed located permanently in one place, but applicants shall be allowed, as far as possible, to choose a locality convenient to them. Nurses must apply to the Hon. Secretary, The Hospital Convalescent Fund, 428, Strand, explaining their inability to secure a needed change without the help of the Fund. Urgent cases will receive the first consideration.

### MISSING NAMES.

Again we have to reproach several correspondents with sending their contributions in without names or addresses attached. It seems strange that readers of The Hospital should take the trouble to write letters, often very interesting ones, and omit to note our rule against inserting anything which is not accompanied by the sender's name. This need not necessarily be printed, but it must be sent to us, and we shall be very glad when our contributors allow themselves to be convinced of this fact.

### THE PENNEFATHER MEMORIAL.

The Invalid Home at Mildmay Park has been formally dedicated and opened. It consists of the two houses which were originally inhabited by Mr. and Mrs. Pennefather, and they have now, by means of certain alterations and additions, been adapted to their present purpose. The invalids which it is designed to shelter will be principally workers who have "given their lives and their strength to the work of Mildmay either at home or abroad."

### HELP FOR THE HELPLESS.

The society formed a few months ago for the purpose of obtaining proper attendants for the aged and infirm inmates of workhouses is now prepared to receive applications from women of good character. This, we may remind our readers, is an altogether new departure, and it aims at obtaining kindly care for the helpless poor in the workhouse wards, not in the workhouse infirmaries. In the latter fully trained nurses are by slow degrees finding admittance and constant employment, but the infirm and aged also require tender, skilful, intelligent care, and infinite patience. Hitherto most of them have been left to the mercy of the so-called able-bodied paupers, who are frequently as incompetent and often but little less feeble than their charges. The committee, which includes Lady Meath and Miss Louisa Twining, can be addressed by applying to the Hon. Secretary, Miss Lee, 20, Bryanston Street, Portman Square. We hope that it will be found practicable to offer fair wages to suitable persons, as the £23 per annum spoken of in Lady Meath's letter to the *Daily News* will hardly attract applicants who, presumably no longer in their first youth, need themselves to make a provision for the future. The work of conscientious workhouse attendants will be incessant and onerous, and if they are to be of the right type, they should be offered higher pay than that which is easily earned by a young housemaid in a comfortable and easy situation.

### IMPROVEMENTS AT WIGAN.

The Board of Management have unanimously adopted the report of the Building Committee at the Royal Albert Edward Infirmary, Wigan, and there appears every prospect of the proposed improvements being shortly commenced. These include a new nurses' dining hall, the present one being in every way unsuited for the purpose, and nurses' quarters, sitting-room, &c. The committee are to be congratulated on their united action, and we cordially endorse their remark that, "In order to secure and retain the services of better class nurses it is imperative that a bed-room be provided for each nurse."

### FAITHFUL SERVICE.

In April the annual meeting of the Kent Nursing Institution for Hospital-trained Nurses took place at West Malling, the Dowager Viscountess of Aylesford presiding. The Lady Superintendent, Miss Tigertwood, has been presented with a silver-gilt medal and clasp, "as a slight recognition of her services to the institution during the past eight years." Nurses Binns and Piggott received a silver medal and gratuity of £10 each for devoted service during the same period, and Nurses Broughton and Griffiths were presented with bronze medals at the expiration of four years' service. This institution is certainly to be congratulated on its work and its workers.

### A SUCCESSFUL BAZAAR.

A bazaar was held last week at Owens College, Manchester, in aid of the funds of the Southern Hospital, and in the course of the three days' sale £2,490 was raised for this excellent object. The new buildings, as well as the amalgamation arranged with St. Mary's Hospital, will enable the good work of the Southern Hospital to be on a considerably increased scale, and the women and children admitted there will undoubtedly benefit correspondingly. The kindly interest of the authorities of Owens College in the bazaar was pleasantly expressed by Principal Ward, who opened it on Saturday, and who also specially referred to the beautiful custom of calling institutions for the sick by the expressive title of hospital. The Southern Hospital is certainly to be congratulated on the increased facilities for exhibiting hospitality which it will for the future enjoy.

### THE BERWICK LADIES.

At the adjourned meeting of the Berwick Ladies District Nursing Association, the question of gentlemen being admitted to membership was discussed at some length. The association has become a flourishing one under the management of the ladies, and the question is now raised as to whether male subscribers of five shillings shall be countenanced and allowed to vote at general and annual meetings. As all those present at the meeting seemed agreed as to the organising abilities of the ladies, there appears no need for the management of the association to be in any way altered, and it is certainly a compliment to present efficiency that the doctors and clergy should wish to have their names added to the Berwick Nursing Association.

### GOOD WORK AT TAUNTON.

THE rules of the Taunton District Nursing Association, as shown in the printed report, appear to be excellent ones, and the amount of work done amongst the sick poor is exceedingly valuable. The Society was first started two years ago in a very modest way, but the hearty support which it has received has enabled it to greatly enlarge its field of usefulness. The addition to the staff of a qualified maternity nurse has worked most successfully, and the women she has attended have done well and seem to have highly appreciated her skill and kindness. A Samaritan fund has enabled the committee to relieve many urgent cases of distress without trenching upon the income of the Nursing Association, and useful gifts for patients have been received from various sources.

### QUEEN'S NURSES IN WALES.

THE annual meeting of the Welsh branch of the Queen's Jubilee Institute took place recently, and a very satisfactory report of the work and finances of the association was given. Attention was called by one of the speakers to the fact that the Cardiff "Hamadryad" hospital ship was without nurses, the sick sailors being left in the hands of untrained men, which, he considered, as reported by the local press, "a disgrace and a stigma which should be removed." At present there are six nurses in the Home at Cardiff, and three will remain on the permanent staff, whilst the others, after the completion of their district training, will be passed on to fresh places, new probationers succeeding them in the Home. The Guardians have decided to have all their outdoor paupers who require skilled attention put into the care of the Queen's nurses, and have made a grant of £50 per annum to the association.

### LOVING SERVICE APPRECIATED.

THE Lady Superintendent of the Perth Sick Poor Nursing Society received a pleasant proof of the regard in which she is held in that neighbourhood, on her universally regretted resignation of her work in the north. The committee and others interested in the association presented her with a handsome tea service, on the tray of which was engraved, "To Sister Bowlby, from friends in Perth, in grateful remembrance of pleasant intercourse, and five years of loving service among the sick poor."

### KINDLY CO-OPERATION.

THE ninth annual report of the Perth Sick Poor Nursing Society, which is now affiliated with the Queen's Jubilee Institute, has recently been issued. The contribution of 217 new garments by the Ladies' Needlework Guild was gratefully acknowledged, such gifts being particularly acceptable. Another useful auxiliary of the nursing society is that which supplies soup daily to the sick poor, a number of ladies having arranged to have it ready on succeeding days. Both these movements are good evidence as to the cordiality with which the work of the Queen's Nurses is supplemented by the residents in Perth and its neighbourhood.

### THE CEYLON NURSES' ASSOCIATION.

A MEETING of this association was held at Nuwara Eliya, on March 27th, and Mr. Brine opened the proceedings in the unavoidable absence through illness of Lady Havelock, who was to have presided. Mrs. Pole-Carew gave an address and explained the scheme originally started by Mrs. Baker and herself for supplying English-trained nurses to the Europeans in Ceylon. Two nurses are already at work, a third is going out this month, and, if the funds permit, a fourth will be secured early next year. Even four nurses are, however, considered quite inadequate to meet the constant demands of the English residents for skilled attendants. The establishment of a hospital in connection with the Nurses' Home has been suggested, but for that, and for an increased nursing staff, the funds need to be greatly augmented. Mrs. Pole-Carew has been appointed Hon. Secretary and Treasurer to the association, in connection with an influential local committee.

### STRENGTH USED NOT WASTED.

"IT'S an odd thing that people always think that it's what you do for others that tires you," remarked an observant nurse, "it's never what you do for them individually." And truly a patient is apt to imagine his nurse tired because her last case was a trying one, or on account of some walk she has taken, or from dusting the adjoining dressing-room, or such work as he, not seeing, considers superfluous. The "friends," perhaps, own that the patient exhausts the nurse's strength, but seldom realise how much their own thoughtlessness may add to her work. And the nurse? Well, doubtless she sometimes fails to judge fairly as to cause and effect, and thinks that all fatigue comes from without, yet much of it might be avoided by intelligently economising strength on occasions when doing so can in no way interfere with duty to the patient.

### SHORT ITEMS.

AT the annual meeting of the Hanley Nursing Society a record of useful work was shown, and the fact that the society was now worked on unsectarian principles was pointed out.—A donation of £500 has been anonymously given to the fund for providing a home for the Wolverhampton branch of the Queen's Jubilee Institute.—A lecture on "Healthy Homes" has been given in Melbourne by Dr. Gresswell, medical expert to the Board of Public Health.—At a recent quarterly meeting of the Stockton and Thornaby District Nursing Association, a report of the work accomplished by means of the temporary convalescent home was noticed. This Home (Sadberge Hall) was used for twenty-three weeks last year, and received in all seventy-four women and children.—*Nursing News* for May contains notes of a lecture given by Dr. Savage at the Midwives' Institute; the first of a series of articles by Miss Catherine J. Wood, and many other papers of interest.—The sale of work in aid of the District Nurses' Fund, held in Hadleigh Town Hall, realised about £30.—It has been arranged to add three probationary nurses to the staff of the Steyning Workhouse Infirmary, and the necessity for providing a nurses' bath-room in the new building has been urged upon the Works Committee.—Mrs. Caroline Pratt has been appointed workhouse nurse at Huntingdon.—The Camborne District Nurse Association has held the fifth annual meeting and show a record of good work.—A branch of the Queen's Jubilee Institute is being formed at Pollokshaws.

### On General Nursing.

By Rowland Humphreys, M.R.C.S., L.R.C.P.Lond.

X.—PNEUMONIA (*continued*).

The crisis is one of the most striking events in pneumonia. It is the abrupt termination of the severer symptoms of the complaint, combined with the fall of the temperature. The latter may fall to normal in some sixteen hours from the commencement of the crisis. This sudden ending of the complaint is often rendered still more striking by the fact that the fever often runs up to a great height just before it takes place. Other things which send the fever up, and must not be confounded with the crisis, are menstruation and the appearance of death. The crisis commences usually by the patient falling into a deep sleep from which he wakes up with a moist skin, a loose cough, and increased expectoration; the pulse, which has been hard all through the complaint, becomes soft, and may become irregular. The bowels may become relaxed, and the flow of urine becomes freer.

The lung symptoms may show no improvement in spite of the other good signs, and the dulness may even extend.

Sometimes instead of the acute symptoms terminating in this rapid manner, the illness may end more gradually and imperceptibly. This is called ending by lysis.

When the lung begins to show signs of improvement it means that the clinical stage of resolution has been reached. In this disease, it has to be borne in mind that the changes in the appearance of the lung do not entirely coincide with the physical signs, or rather, that in several of the stages noticed on post-mortem examination there has been no such definite changes as one would perhaps have expected capable of being recognised by the touch, ear, temperature, or pulse. Thus, although the attempt has been made in this article to make the description of the clinical stages coincide as far as possible with the pathological ones, yet overlapping will occur.

The stage of resolution is shown, on microscopical examination, to mean that the solid material in the lung cells has undergone fatty degeneration, so that it can now be either expectorated up, as happens to a very small extent, or may be reabsorbed into the circulation. The pressure on the vessels being taken off, the circulation recommences to run in its ordinary manner, and the blue appearance of the patient rapidly passes off. The lung may take weeks before it has recovered its normal condition, the dulness on percussion remaining for a long time.

The pleuritic effusion is usually rapidly absorbed as the lung clears up, but it may pass on to form an empyæma, pus being secreted. The most serious danger connected with this stage is the weak condition of the heart, especially as the patient begins to move about more, and the end of the first week is the time when failure of the heart most often occurs.

The pulse gives warning of the heart's condition. It becomes irregular, quickens, or intermits. During the height of the disease it runs a parallel course with the temperature; any change, therefore, either at or after the crisis, or during the fever, indicates danger of failure of the circulation.

Dyspnœa is one of the troubles of this complaint. It may be due to the fever, or to cardiac failure, or to malæration of the blood. It may also be due to pleuritic effusion compressing the lungs. It has to be treated by stimulants, &c., according to the cause.

Death results from failure of the heart, or from œdema of the affected lung. The signs of the onset of the former is mentioned elsewhere. Those of the latter consist in increased and watery secretion in the bronchial tubes, as shown by free watery expectoration, and an increase in the rales, and with these go the signs of impending asphyxia.

Failure from general weakness due to the general condition is shown by the tongue becoming dry and cracked and brown, the temperature falling, the extremities becoming cold, and free perspirations appearing. In intemperate people a high temperature is of very bad omen. Death may also occur from the shock of the onset of the fever, or from excessive rise of temperature. In the first case nothing can be done; in the second the treatment must be directed to lowering the fever and keeping the circulation going.

Some of the complications of acute pneumonia have already been enumerated, but besides these bronchitis may come on, especially in the young and in the aged. Hot, rather than cold applications are required in bronchitis, and expectorants are indicated.

*Pleurisy*—The symptoms due to the inflammation of the pleura are pain in the side, increased on breathing or respiration so thatthe patient holds his breath and fixing the affected side moves it as little as possible; on ausculating, or listening with the ear to the chest wall a rubbing sound will be heard, from the surface of the pleura being roughened by the stripping off of its epithelium, or surface, and the deposit of fibrin in its place. The two surfaces of the pleura are now no longer smooth and polished, and the sound heard is that due to the friction between them. The pain is due to the same cause, and the stitch often complained of is probably caused by the spasm of the intercostal or rib muscles, in their sympathetic endeavour to prevent the movement in breathing which increases it so greatly. Pressure on the affected side relieves the pain and also tends to prevent movement, so the patient usually lies on his bad side; this can be imitated by pressure with bandage, but the obstruction to respiration from the pulmonary inflammation usually prevents this last from being long continued as the bandage compresses both sides of the chest. Strapping acts better in this respect, but often irritates the skin. The strapping is put on in pieces one and a-half or two inches broad, and applied from spine to sternum, beginning at the lower part of the ribs and making one piece overlap the other, just as in the case of broken ribs.

Hot soothing applications such as laudanum or poppy head fomentations sometimes relieve. Occasionally in strong full-blooded persons leeching may be ordered, from six to twelve being used. They may be got to bite, if there is any difficulty about it by putting a little milk on the skin and covering them by a cup or saucer. A large poultice is applied afterwards, the effusion is poured out, and free bleeding allowed. In a few hours there is a free flow of fluid into the pleural sac, and the surfaces being separated by it no longer rub against and irritate one another. The danger of too free effusion then comes on and the nurse has to be aware of an increased amount of rales in the opposite lung and free watery expectoration, which with blueness of the lips or finger-nails, failure of the pulse, tendency to fainting, coldness of the extremities, and deficient flow of, probably, albuminous urine tend to show that the obstruction to breathing has reached the stage of causing œdema of the working lung, and deficiency in its breathing power, with too great strain thrown on the heart and impending failure of that organ. The chest has to be tapped, under the strictest antiseptic precautions for fear of the fluid becoming purulent, and a large amount of fluid may be drawn off without causing untoward results, though there is a tendency to fainting, and the displacement of the heart if too suddenly remedied may even kill the patient.

Pericarditis is sometimes seen as the result of an extension of the inflammation from the pleura.

Jaundice, in a slight degree, is common. It may be due either to extension of the catarrh of the duodenum (nearly always present in these cases) up the bile duct, or from the hepatic congestion due to the impeded circulation.

Cerebral pneumonia, as it is called, means merely severe headache or active delirium, though it is remarkable how often the head symptoms recur, and recur with fresh extensions of the lung inflammation. They generally precede it, and give the impression that there is a very close connection between the circulation to the brain, and, as a part of it, to the respiratory centres, and the inflammatory exudation into the lung; as if the injury to the brain resulted in the congested condition of the lung.

Another complication is gangrene of the lung, due to the defective blood supply to its tissues, as well as to the ingress of special germs. It is recognised by the horrible fœtor of the breath, and is a hopeless sign.

(*To be continued.*)

### The Unveiling of the Memorial to Mrs. Wardroper.

### AN IMPRESSIVE CEREMONY.

The fine chapel of St. Thomas's Hospital, London, was quite full on Monday, April 30th, when the Lord Archbishop of Canterbury unveiled the memorial to the late Mrs. Wardroper, for 33 years matron of this hospital. Amongst those present were: The Treasurer (Mr. Wainwright), the Treasurer of Guy's Hospital (Mr. Lushington), Mr. Rathbone, M.P., Mr. Bonham Carter, Mr. Burdett, Mr. Croft (the consulting surgeon), several members of the medical staff, Miss Gordon (matron), Miss Vincent, Miss Rosalind Paget, Mrs. Dacre Craven, Miss Hughes, and a large number of members of the Nightingale School past and present. It was pleasant to notice that the assemblage included so many of the older governors and members of the medical profession who had had the privilege of working with the late Mrs. Wardroper. The short memorial service included three hymns from "Hymns Ancient and Modern," Nos. 274, 428, and 166; three collects, Psalm 34, and a special lesson taken from the 10th chapter of St. Luke, verses 25 to 38, which was impressively read by the Treasurer.

Mr. Bonham Carter, secretary of the Nightingale Fund, explained the object of the Memorial, and gave a short sketch of Mrs. Wardroper's career, a full account of which appeared in The Hospital of December 31st, 1892, and of January 7th, 1893. Mr. Bonham Carter specially dwelt upon the foresight and co-operation of Mr. Baggallay and Mr. Whitfield, who were respectively Treasurer and Apothecary to St. Thomas's Hospital in 1854, when Mrs. Wardroper was first appointed Matron. The late Drs. Peacock and Bernays, with Mr. Le Gros Clarke, were the most active members of the medical staff when the hospital was removed to the old Surrey Gardens. Mr. Carter especially emphasised the fact that there was no training for nurses at the hospital until the establishment of the Nightingale School in 1860, of which Mrs. Wardroper became the first Lady Superintendent. Mrs. Wardroper's interest in her work was unbounded, her energy was immense, and her devotion extreme. Miss Nightingale had thus described Mrs. Wardroper's personal characteristics:

### Miss Nightingale's Tribute.

It is difficult to describe the character of such a woman, the more so as her praises were never sounded in newspaper or book. No laurel encircles her brow, no crowds assemble to do her honour. But hundreds of trained nurses and hundreds of thousands of well-nursed sick are her meet tribute. Her power of organisation and of administration, her courage and her discrimination in character were alike remarkable. She was straightforward, true, upright. She was decided. Her judgment of character came by intuition—at a flash. Not the result of much weighing or consideration. Yet she seldom made a mistake. And she would take the greatest pains with her written delineations of character required for records, writing them again and again, in order to be perfectly just, not smart or clever; but they were in excellent language. She was free from self-consciousness. Nothing artificial about her. "She did nothing because she was being looked at, and abstained from nothing because she was looked at." Her whole heart and mind, her whole life and strength were in the work she had undertaken. She never went a pleasuring, seldom into society. Yet she was one of the wittiest people one could hear on a summer's day, and had gone a good deal into society in her unmarried life. She was left a widow at forty-two with a young family. She had never had any training in hospital life. There was none to be had. Her force of character was extraordinary; her word was law, for her thoughts, words, and actions were all the same. She moved in one piece. She talked a great deal, but she never wasted herself in talking; she did what she said. Some people substitute words for actions, she never. She knew what she wanted, and she did it. She was a strict disciplinarian; very kind, often affectionate, rather than loving. She took such intense interest in everything, even in things matrons do not generally consider their business, that she never tired. She would be quite late in decorating the chapel at Christmas and Easter with her own hands. She had great taste, and spent her own money, She was a thorough gentlewoman — nothing mean or low about her—magnanimous and generous, rather than courteous. And all this was done quietly.

Mr. Wainwright, the Treasurer, returned the thanks of the Governors to the Archbishop for his attendance that day. He said the late Mrs. Wardroper was a faithful servant of God and man. He had had the privilege of working with Mrs. Wardroper for sixteen years, and so had acquired a full opportunity for judging her life's work. Such a woman holding the position she did in 1860 made it possible to establish the Nightingale School upon the soundest basis, and so to ensure the far-reaching results which that school had achieved in the best interests of humanity and sound nursing. It was the mother of trained nurses. Mrs. Wardroper had had two worthy successors in Miss Pringle and Miss Gordon, the latter of whom was a tower of strength to the hospital and the home at the present time.

The Archbishop of Canterbury then unveiled the memorial, and read the inscription, which we published in last week's issue. The Archbishop then said: This memorial we dedicate to the honour of Almighty God, in memory of Sarah Elizabeth Wardroper. In the name of the Father, and of the Son, and of the Holy Ghost. Amen.

### Address by the Archbishop of Canterbury.

It is probably impossible for the majority of the present generation to estimate at its true value the work done by two noble women, Miss Nightingale and Mrs. Wardroper. The nursing and general efficiency of the hospitals at the present time are so uniformly good, that very few now living can appreciate the difficulties which beset Mrs. Wardroper forty years ago, when nurses were untrained, and were mostly drawn from the lower strata of society. At that time, as a young clergyman, he was in Rome, and had many conversations with a great and intelligent layman of those days about the Crimea and the war. He dwelt with special commendation upon the devotion displayed by the band of thirty-seven nurses who went to the seat of war under Florence Nightingale. The layman expressed surprise that the Archbishop should hold such strong views on the point, and added that without an organisation of this kind he could scarcely think that the claim of the Church of England was high. He, the Archbishop, felt it a privilege to bear testimony to the enormous influence for good which the work of Florence Nightingale had had on the Church of England as a striking fact. He remembered the sensation created in London on receipt of the news that this devoted band of nurses had landed in the Crimea on the day of the battle of Inkermann. History would do justice to Miss Nightingale's vigour, great judgment, and mastery of her subject. The masculine power with which she expounded her plans to statesmen and generals was remarkable, whilst her gift of administration soon produced discipline, comfort, and sanitation amongst the sick. Nor was this all, for the moral influence of her work speedily made an impression upon the people at home. Its effects were remarkable, they produced a revolution in the care of the sick, secured the intelligent co-operation of hosts of good women, and were felt throughout the whole of the British Empire. It was fitting that St. Thomas's

### THE MEMORIAL TO MRS. WARDROPER.

**Anyone wishing for Copies of this reproduction, printed on tinted paper, should apply at once to The Scientific Press, 428, Strand, who will supply copies at Two Shillings each.**

Hospital dedicated to St. Thomas-à-Becket with its venerable history, should worthily become the pioneer in this crusade on behalf of the sick. As Archbishop of Canterbury he felt that St. Thomas's Hospital came home when it came to Lambeth and occupied a site within view of the White Tower of Lambeth Palace, which contains Thomas à Becket's effigy. It was a pleasure to come over to this great building, which he saw every day, and to speak to the memory of her who was preparing for Florence Nightingale's work at home during those fateful months in the Crimea. How great was the blessing upon Mrs. Wardroper's work, who in quiet resourcefulness was reforming and conforming nursing, and preparing the way for better things, until when the nation awoke to the character of Florence Nightingale's work, and subscribed £50,000. St. Thomas's Hospital was ready to make that memorial a practical success.

What a blessed career, indeed, was that of Mrs. Wardroper, who, with all her heart, devoted the best years of her life to great works of mercy and loving tenderness to the sick. How blessed is the work of such a woman, how far-reaching its results, how great its usefulness, with 500 nurses and 50 matrons trained by her own master hand, and distributed throughout the world to act as centres of training and distribution for thousands of other attendants upon the sick. How simple and loving was the nature which led her, after the toil of the day, to spend hours in decorating this very chapel, which she loved so well, for festivals and high days. Here, too, she herself by this memorial will be decorated and commemorated for ever. Her nurses and their successors will pass and repass in and out of this place for ever, attended by the blessings of dying men and the gratitude of the living. Edward VI., on his death-bed, said: "Lord, I thank Thee that Thou has given me life thus far and thus long to finish this work to the Glory of Thy Holy Name!" So we will part with her we commemorate to-day, who might have repeated as her last words those uttered by Edward VI., and say with Florence Nightingale: "And so, dear matron, as thou was called so many years, we bid thee farewell, and Godspeed to His higher world; not as the world giveth, giveth He thee."

The Archbishop then pronounced the Benediction, and thus concluded a most affecting service, which was worthy alike of the occasion and the woman commemorated.

### Appointments.

[It is requested that successful candidates will send a copy of their applications and testimonials, with date of election, to The Editor, The Lodge, Porchester Square, W.]

King's County Infirmary.—Miss Anna Hartford has succeeded her sister as Lady Superintendent of this infirmary. She was trained at Dr. Stevens' Hospital, Dublin, and remained there two and a-half years, and was afterwards Head Nurse at St. Mark's Ophthalmic Hospital in the same city. We congratulate Miss Anna Hartford on her appointment, and wish her success in carrying on the work which has been so efficiently organised by her sister.

### Presentations.

A handsome silver tea-set has been presented by the surgical and nursing staff to Miss G. R. Hartford on the occasion of her resignation of the post of Lady Superintendent of King's County Infirmary, Tullamore. The servants at the institution gave a charming silver butter-dish, and many congratulations and good wishes on her approaching marriage accompanied the gifts to Miss Hartford.

### Our Paris Letter.

French Depopulation.

The question of the depopulation of France has attracted considerable attention in the medical world of Paris. Dr. Tarnier, the well-known gynecologist, has offered a prize of 100 francs (£4) to every mother who is delivered of twins in his lying-in wards. Dr. Budin attacks this important question in another way. He has organised a system of free consultations for the benefit of the infants born in his wards at the Charité Hospital. Every Friday morning he and Dr. Chavanne devote several hours to the examination of from forty to fifty infants. The names of these children are inscribed on a blackboard. They are weighed, and the result written on a card bearing the child's name, the date of its birth, its allowance of sterilised milk, in most instances where the child is brought up entirely by hand, partly by hand, or on mother's milk, supplemented by the sterilised product. It is thus easily ascertained whether the babes increase in weight or decrease, and also whether the ration of milk should be increased, or some other form of food added. Notes of the progress of each infant from birth are carefully tabulated by Dr. Chavanne. To ensure the proper nourishment of his juvenile out-patients Dr. Buden has, at his own expense, arranged a laboratory where milk is sterilised for their consumption. Every morning the precise quantity of milk needed for the daily supply of the children is sterilised, the milk being analysed by the chief analyst of the hospital. The sterilisation is effected by an ingenious apparatus constructed by M. Gentile. Dr. Buden's maternity wards furnish interesting data respecting sterilised milk as an article of food for new-born babes, showing its great value. At the head of each bed is a card showing the range of the mother's temperature and the weight of the infant. Many of these cards prove that the infant has regained its initial weight on the fourth day, though the ninth day is the more usual for this occurrence. Dr. Buden and Dr. Chavanne administer sterilised unwatered milk to the infants as soon as they are born, and continue this system until the mother has sufficient milk to nourish her child and ensure its healthy development. The results are eminently satisfactory.

### Hospital Treatment.

Miss Beatty has written to us a very long letter giving her explanation of the circumstances under which she was admitted to and discharged from King's College Hospital. We are unable to publish it in full, but subjoin extracts from it replying to the statement of the hospital authorities published in our issue of April 28th:—

It is true I entered the hospital under the assumed name of "Court," the assumption being known to the doctor who had attended me for months, and also to my solicitor, for the reason stated in your notice. You say that I brought an action against a doctor to get damages "for an operation performed without my consent." It is wrong; the action tried was for "false imprisonment," &c. The medical point and dispute was not tried, nor has it been settled. It is also true that to hide my personality I gave my occupation as "Governess," that being my means of livelihood before entering the nursing world. Thursday morning, after a quarter to six breakfast, with the knowledge of being homeless (after asking the porter when I could see the secretary, and being informed at half-past nine), I went out to Covent Garden post-office to try to get a telegram through to my sister, but failing, wrote a letter, and being in pain sat by the post office. This was the business which took me out so early; there was no objection raised, and it was because I was so ill and in pain that I asked, nay, begged, for re-admission. Relating to the policeman, it was because I was "in pain, hungry, and almost dead from tiredness" that the inspector humanely sent a constable with me to Bayswater to save me from waiting longer and suffering unnecessarily. I have again to go into hospital.

### Women's Work.

### WOMEN AS HORTICULTURISTS.

We have heard, and still hear, a vast amount of talk on all sides as to a "Woman's sphere." Now, what is a woman's sphere? That question has been asked many times, and answered in many ways; it depends also very much upon who answers it. At any rate we may conclude by their success in it that horticulture may well be reckoned as women's work, and an increasing number of them are taking it up year by year.

The one thing needful, or, perhaps, to be more correct, one of the things needful, is strength. Not that it requires a veritable giantess; but at the same time a really delicate woman should not undertake this work, although for anyone who has no definite ailment, even if not very strong, no occupation is more healthy. It is surprising how quickly women get hardened to working in all kinds of weather; but, nevertheless, unless there is natural love for it, gardening may be found to entail a great deal of pure and simple drudgery.

Patience, perseverance, and energy are three most necessary qualifications. Patience is required, for crops will not grow all at once; they very frequently fail, and then the gardener has to persevere with another, and yet another, waiting and watching to see how they in their turn are going to develop. The horticulturist must be about very early in the mornings to pack, or superintend the packing, of the wares sent away to the markets by the early morning trains, and this involves a great deal of self-denial and perseverance.

Many ladies are now engaged in this branch of work in England, and they often receive pupils to train. The Women's Gardening Association, in Lower Sloane Street, takes pupils as apprentices. At the woman's branch of the Horticultural College at Swanley, in Kent, the teaching is both scientific and practical. Pupils are qualified to hold appointments as market gardeners, head gardeners on private property, under gardeners, specialists, lecturers, practical teachers of horticulture, &c.

The methods of instruction at Swanley embrace class teaching and practical work, the former including the study of horticultural, agricultural chemistry, geology, entomology, and botany. The practical instruction consists "of the demonstration and exposition of the best methods of procedure, so as to illustrate the connection between the science and the art of horticulture." This, of course, includes practice in the arts of flower, fruit, and vegetable culture under glass or out of doors, the treatment of seeds, the propagation of plants, the preparation of soils, and the training, budding, and grafting of fruit trees.

Another part of the work which is of supreme importance to the practical gardener is the buying and selling of produce, the packing and marking of fruit, the storing and preserving of flowers, fruits, and vegetables, including the studies of book-keeping, surveying, and construction of buildings. Other subjects of practical demonstration are poultry-keeping, dairy work, and bee culture. From all this it will be seen that the work is practical, varied, and interesting, without the monotony that is to be found in so many other occupations.

Finally, as regards cost, the committee of the Swanley College have two pleasant semi-detached villas, only a few minutes' walk from the College itself, for the boarding of students, and the cost of residence there, including college fees, is from £70 to £80 per annum. The College course is supposed to take about two years, and those who are successful in passing a somewhat stiff examination receive the College diploma. Examinations are held at regular intervals, and a certificate may be obtained at the end of one year, but the diploma can only be gained after two years' theoretical and practical work. The College receives an annual grant from Government, and two or three years ago the Kent County Council determined to devote a portion of their Technical Education Fund to provide scholarships for the sons of parents of limited incomes. Now five scholarships of £60 a year have been extended to the women students, besides other scholarships of the value of £30 per annum and one of £25, which is given by the committee of the woman's branch.

The prospect of getting a good appointment at the end of the College training appears encouraging for those who obtain diplomas, for it seems that there is still one branch of women's work not as yet overcrowded.

### The Ethics of Nursing.

By Louis Vintras, M.B., B.Sc., Licentiate of the Royal College of Physicians, Member of the Royal College of Surgeons, Resident Medical Officer to the French Hospital.

### III.

Another process that is most objectionable to a sick person, is that of spying. Watch a patient openly, let him know he is watched, the nurse is there for that; but don't lay traps to find him in fault. When he is in fault never screen him, by screening him you become his accomplice, and if it is true that familiarity breeds contempt, it is equally true that the division of a fault's responsibility is not conducive to mutual respect between the two faulty agents.

In all the little cares you have to render to your patient go about them boldly, resolutely, with a purpose; if you bungle, or hesitate, or fuss, you will make him think that you are either afraid, ignorant, or doubtful as to the necessity of your act.

So the following may be given as representing the seven cardinal virtues of the perfect nurse: Obedience, watchfulness, precision, gentleness, abnegation, moral courage, and moral charity.

The gift of conversation was bestowed on man to make weary hours less tedious. This truth is one that meets with early recognition in all persons who have gone through a serious illness, and they have a remarkable propensity for putting this theory into practice, and this propensity is one of the greatest dangers that awaits the nurse. Conversations are inocuous in direct ratio to the number of persons present; the lowest quorum necessary for the transaction of a satisfactory conversation being two, that number is the most dangerous. It is so because conversations between two persons have a disagreeable tendency to assume a confidential character, and confidences from nurse to patient may become, to say the least of it, delicate. Patients are wonderful questionists; this you will find to your cost, and they will make you say some very awkward things about that one great event of their present existence—their disease.

It is well for nurses to realise from the outset that their position is one of great responsibility. Responsibility has its charms; it has also its dangers Of the first I need not speak, of the second I should like to say a few words. Courage is often only the mere habit of danger. To the nurse is left the care of administering the drugs, and it is one of the items to which too much attention cannot be given. It is so easy when in a hurry to mistake one bottle for another, or to give an overdose, or to forget a bottle within reach of the patient. It must be remembered that it is an unwritten law in medicine that the patient has no responsibility, and it is always those in attendence on him who will be blamed for any mishap, the nurse among the foremost. The same zealous care must be extended to dressings; a slovenly or hasty dressing may be injurious, and will always be uncomfortable. Comfort is a powerful aid to the art of healing. It should be borne in mind that in the absence of the medical man the very life of the patient may depend on your watchfulness, on your quickness of observation, and on your experience.

I have said enough I think to give some idea of what may be called the ethics of nursing, and to impress on all a sense of the exacting duties and responsibilities appertaining to one of the noblest careers to which a woman can devote her life.

### Everybody's Opinion.

[Correspondence on all subjects is invited, but we cannot in any way be responsible for the opinions expressed by our correspondents. No communications can be entertained if the name and address of the correspondent is not given, or unless one side of the paper only be written on.]

### THE ROYAL NATIONAL PENSON FUND FOR NURSES.

"Policy Holder 370 and 1,240" writes: As a grateful member of the Royal National Pension Fund for Nurses and sick pay policy holder, permit me to write to my fellow-nurses through the medium of your paper, which has such a wide circulation amongst them, on the subject of the victory of the Royal National Pension Fund for Nurses over the Record Press (Limited). The published apology of the latter should now remove from nurses' minds any false impressions which may have existed previous to this action, and induce them to make provision for their future as soon as possible. During the past sixteen months I have only been well enough to nurse four months, and the greater part of the other twelve I have been in receipt of £1 per week sick pay from the above Fund; still being ill, and certainly shall be under medical treatment some weeks longer, and entirely dependent on my sick pay, as I have no home, my warmest gratitude is given to the founders of the Fund, which proves such "a true friend in need" to nurses, and I am deeply anxious that others should avail themselves of their opportunities to provide for "rainy days."

### HOLIDAY HOMES.

Sister Amy writes: Can anyone give me the address of a holiday home for nurses in Paris? I had a delightful time last summer, for I went with a friend to Tami House, Braintree, which we had seen advertised in your valuable paper. Any information about Paris will be most gratefully received.

### THE JANE H. ORR MEMORIAL HOME, BALLYMENA.

Miss Young, member of the committee of the District Nursing Society, writes: On March 15th the J. H. Orr Memorial Home was formally opened in Ballymena. A preliminary meeting was held in the Home, at which the Right Hon. John Young presided. The Dean of Down offered a dedicatory prayer, and the lease was formally handed to the trustees. Afterwards a public meeting was held in the Town Hall, largely attended by the people of the town and neighbourhood, at which interesting speeches were delivered telling of the quiet work carried on by the District Nursing Society, inaugurated 13 years ago by the late Mrs. Murray and Mrs. King, and under Miss Orr's superintendence it was brought to its present state of efficiency. The latter lady sacrificed her life on behalf of the sick poor, for entering a house where there was typhus fever she contracted the disease to which she succumbed after a few days' illness. The Home recently opened has been erected to perpetuate Miss Orr's memory. The nursing staff at present consists of two Queen's Jubilee nurses, Miss Davison and Nurse Ross, who have carried on the work satisfactorily for the past year, having had 264 cases and paid 8,184 visits.

### TRAINED NURSES' ANNUITY FUND.

Lady Bloomfield writes from 21, Queen's Gate Gardens, S.W.: There have been of late some misapprehensions with respect to the continuance of the Trained Nurses' Annuity Fund, and I have been requested to bring this matter once more before the public. In 1874 a letter from me was published, which was the means of starting an annuity fund for broken-down trained nurses, and at that time this was the first effort made to assist a most useful and deserving class of women, some of whom, after a life of risk and self-denial, were allowed to end their days in the workhouse. I thankfully acknowledge the great efforts which have since been made to remedy this deplorable state of things, especially by the creation of the Royal National Pension Fund for Nurses and the Royal British Nurses' Association. At the same time I wish to draw the attention of the public to the fact that the Trained Nurses' Annuity Fund reaches a class of women who have been precluded by adverse circumstances from insuring their lives in the Royal National Pension Fund, and that it is worthy of a continuance of the support by which twelve annuities have been permanently founded. Most of the original subscribers having passed away, the committee are very anxious to enlist the sympathy of the charitable in a work which has done, and is doing, great good, and which is founded on such a sound basis that it must in time assume much larger proportions than it has at present. Subscriptions and donations will be thankfully received by Messrs. Coutts and Co., 59, Strand; or the Hon. Secretary, Mr. R. Gofton Salmond, 73, Cheapside, E.C.

### ARMY NURSING.

"An Orderly" writes: With your usual courtesy will you allow me to place before your numerous readers a few points in reference to an article in your issue of April 7th, entitled "Nursing of Army Sisters," by A. O. Grey. I don't wish to criticise the statements made by A. O. Grey regarding the duties of army sisters in military hospitals; but I do wish to mention that the asserted non-qualification of the orderlies of the Medical Staff Corps for nursing the sick is very much exaggerated. Summing up the whole article, your readers will naturally think that the orderlies are simply useless, idle, and incompetent, for the writer further states that the Medical Staff Corps chiefly consists of boys with no love for nursing, physically unfitted for any other regiments. Allow me to state that it is no easy matter at the present time to be enrolled in the Medical Staff Corps. Applicants must produce a recommendation from their last employer, and must be of respectable appearance and character. The corps is only open for enlistment during a certain time in each year; and, furthermore, a great many medical students are to be found in this corps. All wards in military hospitals are not sisters' wards, therefore in them it is orderlies alone who are the competent nurses of the patients. Again, who nurse the sick at night, and then have to be on duty all the next day? Orderlies—not the sisters! In concluding may I mention that it is not the sisters but the orderlies who attend on contagious diseases such as scarlet fever, measles, erysipelas. Take some of our foreign service stations again, such places as Singapore and Bermuda, the latter a place where enteric fever prevails, and I think the answer must be that the duties are performed by the orderlies of the Medical Staff Corps. Let it be understood I am simply defending a corps that I am proud to belong to, and one at present put upon and ridiculed by persons who are ignorant of the duties incumbent upon its members.

### DROYLSDEN NURSING ASSOCIATION.

A Correspondent writes: An account appeared in The Hospital of April 21st, taken from a report in the local Press, of a presentation made to "Nurse Mills," in which it is stated that one of the doctors said the ladies connected with the association "interfered too much with the work of the nurse." It is only fair to say that though L. Mills has been working in Droylsden she was quite untrained. A large majority of the committee decided to make a change, and Droylsden is now affiliated with the Jubilee Institute, and one of the nurses has been working for three weeks with most satisfactory results. The paragraph in question is calculated to give a wrong impression, as though the committee had dismissed without sufficient cause a trained nurse, whereas they have now substituted for an unqualified woman a trained Jubilee Nurse.

☞ For Where to Go, Reading to the Sick, Book World for Women and Nurses, &c., see page xlix. et seq.

### The Muses' Looking Glass.

THE NEW GALLERY.—SUMMER EXHIBITION.

THE old order has given place to the new; sweet-faced Madonnas and Holy Families are conspicuous by their absence on the walls of the New Gallery. Last winter's Early Italian pictures exist now alone in our memories, and once more we are rushed into the world of to-day, rudely brought back, as it were, to the present, to the less devotional art of 1894. One walk round the rooms and the depression in trade is perhaps the first thing which shows itself reflected to us in this exhibition. That is, if one may so read the absence of a want of daring in the pictures lining the gallery. Out of the 400 there are few one might classify as "ventures," a precaution consequent on the dearth of purchasers last year for pictures of any large composition. Portrait painters have had the best of it in this summer exhibition. It says a good deal for the personal element of the age to discover no fewer than 81 of these, in various styles and sizes, which are the most striking section among the collection, and they are for the most part conspicuous for their excellence.

Take Hubert Herkomer's "Lady Ridley" for instance (No. 189). It is a life size picture of a lady in light costume, the face and shoulders drawn and modelled in a masterly manner, but the gentle refinement which characterised this artist's "Miss Grant" is painfully absent. The important question of back-ground is perhaps not quite successfully settled in this picture of vast dimensions. The curtain is monotonous, one has an inclination to draw it back, to know what is behind, and there is a look in Lady Ridley's face, too, as if she also shared the spectator's curiosity. In "Nomads" (No. 140), Professor Herkomer has not painted up to his usual mark. It seems likely that Mr. Shannon will bring all things to pass which an earlier estimate of his work led us to anticipate. This year his portraits are especially remarkable. Compare No. 105 and No. 207, hanging in a conspicuous place in the North Room, and the versatility of the artist's scheme of work strikes one forcibly. Composition and technique are alike masterful in both, but Mr. Shannon possesses the further power of adapting himself to his subject. There is no one idea ridden to death in his works, but an individuality of treatment, his style is recognisable by order of its merit, but his portraits are not replicas the one of the other. In his study of Lady Bathurst we find a beautiful, gracious lady graciously and gracefully depicted. Mr. W. B. Richmond shows a fair specimens of his work in his picture of Mrs. R. H. Benson (No. 116), the single instance in which he is represented. Mr. Holman Hunt likewise contributes an isolated example in a small mosaic (may we call it) of Harold Rathbone, Esq. So much for the portraits, of which, however, much is left to be said, for they are, as we have said, the principal feature in the exhibition.

Of composition pictures perhaps the most remarkable is Mr. Matthew Hale's "Mermaid's Rock" (No. 199). This, as its name betokens, is an imaginative subject. It represents a ship on the verge of doom, the sailors, wild-eyed, struggling to turn away from the fatal rock, to which the mermaids have ensnared them. This is a bold-spirited piece of work, full of life, vigour, and action. A particular interest is centred round a replica in oils of Sir Edward Burne Jones's "Love Among the Ruins." Those who knew the original study which met its unlucky fate during a reproduction process in Paris, will be glad to welcome this beautiful piece of composition back to life, but the "inspiration" which prompted the original is not so strongly felt to be present in its successor. Nor has the colouring the same sense of harmony and rest as its predecessor *in tempera*.

As a piece of finely-designed and decorative work Mr. Walter Crane's "Swan Maidens" is perhaps the most conspicuous. This he treats in a fresh and delicate manner, if it fails a little in depth of tone and uniformity of colour. Both landscape and seascapes are generally good throughout the exhibition. No. 210, a monster study of "A Lonely Farm," is depicted by Mr. Alfred Parsons in a new and original treatment of a long stretch of Swede fields. The subject is one dependent on its handling, but the artist has grasped this fact and made of a monotonous landscape a really fine picture. Rustic figures, scattered about, busied in their agricultural pursuits, are bathed, as is sky and land, in the lurid threatening light of a brilliant afterglow. It is a daring subject, but Mr. Parsons has shown himself master of it, and both in theme and execution the Lonely Farm adds conspicuously to the artist's renown. One would have wished for further examples of his work, as also of Mr. Napier Hemy's, who is represented in "Fresh from the Sea" (No. 46), and No. 205, "Cornish Crabbers," an inimitable study of fishing life in full action. The latter of his two pictures is painted in broadly and boldly, and his canvas he fills with a suggestion of life and motion, and surging buoyant waves, which seem to heave and rise as one looks. "The Dawn of the Year," by Mr. George Wetherbee (No. 229), is at best an "attempt," but its conception as its carrying out are weak and affected and beneath the dignity of the subject.

Mr. Edward Stott's "Village Street" (No. 248), is charming and suggestive, the grouping of the half-defined figures in the mid-distance shows the artist at his best. "A Bright Morning After a Breeze" is painted in almost too bright a manner by Mr. Henry Moore—it is too strong in colour to be pleasing, without sufficient depth of tone to be quite natural.

The upper gallery of the exhibition barely repays one the trouble of mounting the steps. One large three-quarter portrait (No. 300), entitled "A Rose," is dignified with "honourable mention," and it is worthy of the honour. The face of the young girl thus depicted has a certain fascination about it, and would have yet a larger hold upon our admiration were her mouth more in accordance with the general charm of the young girl's appearance.

The statuary shows us little that is remarkable. Mrs. J. W. Swynnerton's "Love's Chalice"—a fountain in marble, whereat "Love" holds a cup, out of which a maiden drinks—is the most noticeable. Mr. Onslow Ford's "General Gordon" shows a hero of diminutive size on a camel of equal proportions. Unless the sculptor designed this, as it were, "to space," it redounds but little to his credit. The proportions are too unheroic for the subject it is designed to represent.

### Minor Appointments.

SOUTHEND VICTORIA HOSPITAL.—Miss Ada Britten has been appointed Nurse-Matron of this hospital. She was trained at Hertford General Infirmary, and afterwards worked at the West London Hospital, Hammersmith, and at Andover Cottage Hospital. We wish her every success in her new work.

### Notes and Queries.

The contents of the Editor's Letter-box have now reached such unwieldy proportions that it has become necessary to establish a hard and fast rule regarding Answers to Correspondents. In future, all questions requiring replies will continue to be answered in this column without any fee. If an answer is required by letter, a fee of half-a-crown must be enclosed with the note containing the enquiry. We are always pleased to help our numerous correspondents to the fullest extent, and we can trust them to sympathise in the overwhelming amount of writing which makes the new rules a necessity. Every communication must be accompanied by the writer's name and address, otherwise it will receive no attention.

### Queries.

(53) *Massage.*—Information wanted as to certificates.—R.S.H.

(54) *Ceylon.*—Kindly tell me where I can get information about private nursing in Ceylon ?—*Eleanor.*

### Answers.

(53) *Massage* (*R. S. H.*).—Teachers of Massage give certificates to their pupils for proficiency. There is no one universal certificate.

(54) *Ceylon* (*Eleanor*).—Write to Mrs. Pole-Carew, Hatton, Ceylon, who is hon. secretary to the association, also see paragraph in another column.

### Where to Go.

Hospital for Women and Children, Soho Square.—The Princess Christian will open the new buildings on June 1st.

Trained Nurses' Club.—At 7.45 on Friday evening, May 18th, Surgeon-Lieutenant-Colonel Evatt, M.D. will lecture "On the Organisation of the Medical Service of an Army in the Field." Tickets for non-members 6d. each.

Writers' Club, Hastings House, Norfolk Street, Strand.—This club will reopen in the new premises on May 9th. The President, Princess Christian, has promised to visit the club at half-past three on May 18th to meet the members.

A Missionary Conference of the Anglican Communion will commence on May 28th, at four p.m., with a service in St. Paul's Cathedral, sermon by the Bishop of Durham. The members of the Conference will afterwards be received by the Lord Mayor at the Mansion House. There will also be three sessions each day from May 28th to June 1st in St. James's Hall, Piccadilly.

The Imperial Institute.—The Annual Amateur Art Exhibition, opened on Wednesday, May 2nd, by H.R.H. The Duchess of Albany, remains open till Saturday, 5th, and on that day and the preceding one the entrance fee is only one shilling. The exhibition is in aid of the Parochial Mission Women's Fund, the East London Nursing Society, and East-End Mothers' Home.

"La Société des Acquarillistes Francais." Hanover Gallery, Bond Street.—A small but representative collection of French water colourists is now on view at the above gallery. Taken as a whole these are somewhat disappointing in their want of colour, and vividness. They fall short for the most part in this particular, a failing by no means generally characteristic of the Acquarillistes Francais. The decorative school is represented by G. Rochegosse, who frames his studies in a manner which is wholly original and unique. Nos. 1 and 11 (large processional groups by Bernard) are strong and powerful, and are the principal features of this collection, not by merit alone of their size. Loir Luigi's reputation of grouper and composer is well maintained in his six works here represented. No. 88 is perhaps the best, but they are all studies of massed grouping treated in a consumately clever manner. Portraiture is conspicuously absent. Victor Gilbert's study of a woman's head (No. 59) is bold and life-like. Paul Lecomte's landscape is painted in a clear and decided manner by a hand which has considerable mastery of technique. The same, perhaps, in a greater degree can also be said of No. 122, a sketch by Gaston Roullet, showing in a suggestive way wind sweeping through long grass with the sea in the horizon, dark and sullen, beyond the downs.

### Stockton and Thornaby Nursing Association.

The Marchioness of Londonderry presided at the annual general meeting of the Stockton and Thornaby Nursing Association, held last week in Thornaby-on-Tees. There was a good attendance on the occasion. The co-operation of workmen has been a most satisfactory feature in the organisation of this society, and has enabled it to become a practical and financial success. The Stockton and Darlington Tramways Company provide the nurses with free passes, and the Thornaby Needlework Guild supplies useful garments for needy patients.

### Death in Our Ranks.

We record with regret the death of Nurse Foorte from typhoid fever at the Royal Infirmary of Bristol, which she contracted from a patient in the course of her duties as assistant nurse. She was only twenty-five years of age, but the assiduity and intelligence with which she had entered into her work as a nurse had rendered her one of the most valuable assistant nurses on the staff at Bristol when her career was cut short. The case of enteric fever was of a peculiarly virulent type, and there is little room for doubt that she contracted it from the expectoration of her delirious patient, and not from any want of care in dealing with the discharges. A short funeral service was held in the chapel of the Royal Infirmary on the 24th ult., which was largely attended by the medical faculty, students and nurses, and other friends.

### For Reading to the Sick.

### THE VICTORY.

**Motto.**

Now once more, Eden's door opened stands to mortal eyes,
For Christ hath risen, and man shall rise. —*Neale.*

**Verses.**

Shall man alone, for whom all things revive,
No resurrection know? Shall man alone,—
Imperial man!—be sown in barren ground
Less privileged than grain on which he feeds?—*Young.*

Thou know'st He died not for Himself, nor for Himself arose;
Millions of souls were in His heart, and thee for one He chose,
Upon the palms of His pierced hands engraven was thy name,
He for thy cleansing had prepar'd His water and His flame,
Sure thou with Him art risen: and now with Him thou must go forth,
And He will lend thy sick soul health, thy strivings might and worth. —*Keble.*

Rise, heart! Thy Lord is risen! sing His praise without delays,
Who takes thee by the hand, that thou likewise with Him mayst rise;
That, as His death calcined thee to dust,
His life may make thee good, and much more just.
—*George Herbert.*

**Reading.**

This present life is full of the rythm of the Resurrection; it is ever ready to remind us of the news of Easter. Time after time . . . . we see some rendering, as it were, of the glory of this Queen of Feasts, some parable of the empty tomb and the stone rolled back, and the triumph over death. When the day breaks and the shadows flee away, and all life stirs and wakes again; when the long tyranny of winter yields, and the flowers appear upon the earth, and the time of the singing of the birds is come; when some great sorrow, or anxiety, or mood of sadness passes from our hearts, and we rediscover the reality of joy. . . . When the long days of sickness are forgotten in the new gladness of returning health; the sequence of Holy Week and Easter is enacted, and the note that sounds out loud on that most blessed day is touched again. All life around us and within displays at times some likeness of a rising from the dead.

But in all these types and parables of the resurrection; . . . the brightness is but for a while; the voice of joy and health must fail again, we know, in a few years at the most; the leaves that to-day are just revealing that ever fresh surprise of beauty which will soon be the glory of the spring, must presently be shivering on the trees or scudding along the roads in the November gale; the clouds return after the rain; the morning cometh, and also the night. . . . One alone there is Whose day has no twilight and no night, Whose glory never fades, and over Whom death hath no more dominion, since "Christ being raised from the dead dieth no more." . . .

The crucified declares to us "I am He that liveth, and I became dead; and, behold, I am alive for evermore." Of Himself by the free will of His great love, He laid down His life for us; and now He has taken it again for ever and ever. . . Thenceforth as King and Priest, unchanging and eternal, He ever reigns and pleads for us, in the power of an endless life, an "endless morn of light." . . . But, must we wait until we leave this world to see or know the difference His victory has made? No, we can both see and know it here. It is not far from every one of us. . . . In the character which is formed by Christ's example and sustained by His Sacraments, there is that which is not transient, which being raised from the death of sin, dieth no more.

### The Book World for Women and Nurses.

[We invite Correspondence, Criticism, Enquiries, and Notes on Books likely to interest Women and Nurses. Address, Editor, The Hospital (Nurses' Book World), 428, Strand, W.C.]

A Naughty Girl. T. Ashby Sterry. (London: Bliss, Sands, and Foster, 1893.)

Mr. Ashby Sterry draws his "Naughty Girl" in a most attractive manner, but we find her endowed with other characteristics than that by which her biographer qualifies her. Where indeed does her naughtiness come in? though perhaps, as in other works of fiction, Mr. Sterry names his book to conceal his plot. Unless loyalty and gentleness and domesticity are "naughty," then no more is Beryl Cheyn deserving of her appellation. She does not love bad men nor say smart things. In this necessarily she falls far short of what is expected in female portraiture in 1894. She is not a Dodo or a Yellow Aster, her biographer only aspires to make her a sweet English girl and a decent member of society. She is also behind the times in having no theories—or if she has she is kind enough to keep them to herself. Beryl Cheyn shows herself the right hand and protector of the small sisters left under her charge, and faithful and disinterested in the affection she extends to Mr. Jack Caversham. She lives a quiet monotonous life in Great Ormond Street till an unforeseen occurrence throws her future husband in her path. Between him and the heroine certain troublesome misunderstandings render the course of true love to run in less smooth waters than conduces to the happiness of the young couple. But the end is happy and eventually things clear themselves up. We have known Mr. Ashby Sterry as a writer of society verses and "lazy minstrelsy;" we are glad to find the happy hopeful tone which characterized his rhyme not absent in his prose.

A Superfluous Woman. (London: William Heineman, 1894.)

This is an interesting, albeit impossible story, and the characters and scenes in the book, though well drawn, are somewhat exaggerated. In the first volume we find Jessamine Halliday, the heroine, in a hysterical trance which she has got herself into, apparently in the desire of escaping from ennui and boredom; in fact she is trying a sensation (she is always in search of sensations) which threatens a tragic sequel. She is rescued from this state and restored to reason by a certain charlatan, Dr. Cornerstone by name, who is—although he deems himself a quack—the most charming character in the play. Yet notwithstanding Dr. Cornerstone's kind intentions we feel it might have been better had Jessamine been less impressed by his teachings that made her see for a time the vanities and worthlessness of a society life, for out of it she does infinitely more harm than she did in it, ennui giving place to a fearful energy and excitability which ends in her mysterious disappearance from her home to seek work and strange wisdom in the Highlands. The second volume contains a detailed account of the self-analysis and, we may add, prurient curiosity of the heroine's nature. Colin Magillvray, who now makes his appearance as a Scottish peasant, is a noble character in fiction, though we cannot help a feeling of surprise at his unreciprocating attitude, towards the open advances of so exceptionally beautiful a woman. At the commencement of Jessamine's liking for Colin there is a more wholesome feeling, and a very true touch is brought in when her love for the peasant is banished by the thought of his coarse old father. The third volume shows the result of her philosophy or obedience to society's laws. But here again our incredulity is aroused, and we doubt whether ten years of marriage with Lord Heriot, a dissolute, drunken reprobate, would tend to develop the latent germs of goodness in her nature so as to turn his wife into a forbearing angel of goodness and purity. The scene where she shows Dr. Cornerstone the ghastly outcome of her miserable marriage in the persons of her diseased, idiot children is horrible and realistic, but yet strong and genuine. Death alone can be—and is—the fitting end for "a superfluous woman."

Platonics: A Study. Ethel M. Arnold. (Osgood and McIlwaine and Co., London. 1894.)

This is a characteristically modern literary effort, and the reader is spared none of the elaborate psychic analysis of the female ego which forms at once the nucleus and the terror of Victorian fiction. The tale in itself is simple enough. The three principal actors in it are Susan Dormer, her friend, Kit Drummond, and Donald Gordon, the man whom they both love. How Susan relinquishes her lover to her friend forms the subject of the whole plot. But the interest of the book all lies in the study of Susan Dormer's character. She herself is astonishingly absorbed in her own personality, so absorbed that she allows herself only one friend, Miss Drummond, though in Kit's absence abroad she drifts into friendship with her neighbour, Ronald Gordon, to whom she talks a good deal about herself and her "life current." When he ultimately proposes to her, she says: "Every hour of my life of late years has taught me that form of any kind is the one thing that passes away, that personality, individuality, self, in a word, perishes; that the universal remains." In other words, introspection had resulted in a morbid distaste to her own ego, and a resolution to annihilate it, in which desire she cannot fail to enlist the sympathy of the reader, though she most probably succeeded in boring her lover, for he transfers his affections to Miss Drummond almost immediately after. To the onlooker the character of Susan Dormer must ever remain that of a selfish woman of no interests beyond herself, leading a lonely life, so that when she dies it seems as if her very existence had ceased to be by nature of its own uselessness. However, there is much that is very readable in "Platonics." Two rooms in the old Northumberland Castle are charmingly described; but Miss Arnold outrages all established rules of grammar when she calls the library "liveable." There is a chapter descriptive of a day's salmon fishing, which is very realistic and written in a spirited manner.

### MAGAZINES.

The English Illustrated Magazine for May contains in its first pages a short but pleasing article on "A Premier's Favourite." This is a description of the firm but loyal friendship established between Mr. Gladstone and his small grandchild, Miss Dorothy Drew, "Between," as the writer describes it, "an octogenarian statesman, who has spent more than half a century in moulding the fate of his country, and a tiny girl child to whom life is still so new that the simplest sights and sounds of everyday life are revelations, it might well seem that there must of necessity be a greater, if not an impassable, gulf fixed." Another article reveals to us Robert Louis Stevenson at Valima, Samoa, in an intime and interesting manner. There are besides several short stories, written up to the usual standard one has ever been led to expect in the pages of this magazine. But what perhaps especially appeals to us this month is an article dealing with the old traditions associated with this season. Here we find the customs which still survive and are practised in honour of the Queen of Months traced back to their fountain head, to the primal spirit which dictated the worship. "One cannot," comments our authority, "regard the numerous instances of revived May-day sports in modern times without some feelings of satisfaction, but what is specially to be wished for is a spontaneous revival of these ancient and picturesque pastimes among the people generally, rather than as a result of the patronage of the richer and more influential members of the community."

## Figures and Tables

**Figure f1:**